# The association between organised colorectal cancer screening strategies and reduction of its related mortality: a systematic review and meta-analysis

**DOI:** 10.1186/s12885-024-12054-7

**Published:** 2024-03-21

**Authors:** Hanyue Ding, Jiaye Lin, Zijun Xu, Harry H. X. Wang, Liwen Huang, Junjie Huang, Martin C. S. Wong

**Affiliations:** 1 JC School of Public Health and Primary Care, Faculty of Medicine, The Chinese University of Hong Kong, Room 407, 4/F, Postgraduate Education Centre, Prince of Wales Hospital, 30-32 Ngan Shing Street, N. T., Shatin, Hong Kong China; 2 School of Population Medicine and Public Health, Chinese Academy of Medical Sciences & Peking Union Medical College, Beijing, China; 3 School of Public Health, Sun Yat-Sen University, Guangzhou, China; 4 School of Public Health, Peking University, Beijing, China

**Keywords:** Colorectal neoplasm, Secondary prevention, Early detection of cancer, Mortality

## Abstract

**Background:**

To assess the long-term association between organised colorectal cancer (CRC) screening strategies and CRC-relate mortality.

**Methods:**

We systematically reviewed studies on organised CRC screening through PubMed, Ovid Medline, Embase and Cochrane from the inception. We retrieved characteristics of organised CRC screening from included literature and matched mortality (over 50 years) of those areas from the International Agency for Research on Cancer in May 2023. The variations of mortality were reported via the age-standardised mortality ratio. A random-effects model was used to synthesis results.

**Results:**

We summarised 58 organised CRC screening programmes and recorded > 2.7 million CRC-related deaths from 22 countries where rollout screening programmes were performed. The CRC screening strategy with faecal tests (guaiac faecal occult blood test (gFOBT) or faecal immunochemical tests (FIT)) or colonoscopy as the primary screening offer was associated with a 41.8% reduction in mortality, which was higher than those offered gFOBT (4.4%), FIT (16.7%), gFOBT or FIT (16.2%), and faecal tests (gFOBT or FIT) or flexible sigmoidoscopy (16.7%) as primary screening test. The longer duration of screening was associated with a higher reduction in the pooled age-standardised mortality ratio. In particular, the pooled age-standardised mortality ratio became non-significant when the screening of FIT was implemented for less than 5 years.

**Conclusions:**

A CRC screening programme running for > 5 years was associated with a reduction of CRC-related mortality. Countries with a heavy burden of CRC should implement sustainable, organised screening providing a choice between faecal tests and colonoscopy as a preferred primary test.

**Supplementary Information:**

The online version contains supplementary material available at 10.1186/s12885-024-12054-7.

## Introduction

Worldwide, colorectal cancer (CRC) is the second leading cause of cancer-related deaths, accounting for 9.4% of total cancer deaths in 2020 [1]. As most CRCs may take years to progress from the early adenoma to invasive cancer, cancer screening serves as an effective intervention to remove adenoma and prevent cancer, and further, to decrease CRC-related mortality. According to previous studies, CRC screening with guaiac faecal occult blood test (gFOBT), faecal immunochemical test (FIT), flexible sigmoidoscopy (FS), and colonoscopy could contribute to a reduction of 14–16%, 22%, 28%, and 68% in CRC-related mortality, respectively [2]. Simulation models also showed the cost-effectiveness of these screening tests in comparison with no screening [3]. Therefore, international guidelines recommend CRC screening for older adults aged above 45 or 50 years old [4–6].

The CRC screening involved organised programmes and opportunistic programmes. An organised screening programme includes a systematic process, which is monitored externally at the government or institutional level. Every step that is involved in the organised screening process, from the invitation of the target population to follow-up of screening participants with positive screening tests, is continuously evaluated for quality assurance. Nevertheless, the traditional opportunistic screening approach is largely performed only when a patient visits a physician, which may lead to under- or over-screening, uncertain quality, or health inequity [2]. Levin et al. reported that an organised CRC screening had twice the participation rate (i.e., from 38.9% to 82.7%) when compared with opportunistic screening in California. The higher rates of screening were associated with a reduction from 30.9 to 14.7 deaths/100,000 [7].

A number of organised CRC programmes have been introduced and implemented during the past two decades. A study from Italy contrasted the CRC-related mortality in 2006–2011 with that before screening (1995–2000) and observed a 22% decrease between early screening areas and late screening areas [8]. The average annual percentage changes were employed by Cardoso et al. to compare the epidemiological changes of CRC in relation to screening implementation across Europe [9]. Nevertheless, to our best knowledge, no study has been conducted to systematically summarise the changes in mortality as a result of the initiation of organised CRC screening programmes worldwide.

Therefore, we aimed to systematically review the current organised CRC screening programmes and match their CRC-related mortality before and after the screening implementation. We also assessed the long-term association between organised CRC screening strategies and CRC-related mortality.

## Materials and methods

### Literature search

This study was registered on PROSPERO International prospective register of systematic reviews (CRD42021231274). We systematically reviewed all organised CRC screening through PubMed, Ovid Medline, Embase and Cochrane from the journals’ inception to Jan 2023. Grey literature was examined via hand searching for relevant CRC screening programmes conducted at national levels. Other related reviews involved in organised CRC screening were also included as references. The following keywords and MeSH terms were shown in Table S1. There were no limitations on language or types of publication.

### Data extraction

Two reviewers (JL and ZX) independently performed the literature search, and any disagreements were resolved by a third reviewer (HD). The characteristics of CRC screening was extracted, including countries, screening status, starting age, screening modality, programme duration, screening coverage, and participation. Two independent reviewers (JL and ZX) also retrieved the age-standardised CRC-related mortality (more than 50 years) of countries that conducted organised CRC screening from the cancer mortality database of the International Agency for Research on Cancer (IARC) [10]. CRC was identified as C18-21 in terms of the International Classification of Diseases 10th revision (ICD-10). The CRC-related mortality was retrieved from the screening initiation year to the most recent year with available data (extracted in May 2023). As all mortality data was from the IARC database, no risk of bias assessment was involved in this study.

### Statistical analysis

The variations of mortality were reported, and the age-standardised mortality ratio (ASMR) was calculated by the mortality in the initial year and the latest year. We used a random-effects model to synthesise the ASMR with a 95% confidence interval (CI). I^2^ was reported to evaluate the heterogeneity, and I^2^ of 25%, 50%, and 75% indicated low, medium, and high heterogeneity, respectively [11]. Since most I^2^ of indicators are more than 50%, we did not use a fixed-effects model to pool the ASMR. Subgroup analyses were performed in terms of screening modalities, screening duration, and sex. We classified screening modalities into 5 types according to the primary screening tests: 1) gFOBT only; 2) FIT only; 3) gFOBT or FIT (switching from gFOBT to FIT or adopting anyone at the same time); 4) faecal tests (gFOBT or FIT) or FS; 5) faecal tests (gFOBT or FIT) or colonoscopy offered as options for prospective screening participants.

Since the diversity of screening programmes between countries might have an impact on the mortality outcomes, a sensitivity analysis was performed to test the robustness of the results. If we treated the annual ASMR as longitudinal data, a statistic model should take into account both the changes over time and the correlations between the repeated measurements in each country. A linear mixed model was applied in this study. The dependent variable was annual ASMR, and the independent fixed factor was the modality. The country was a random factor that allowed heterogeneity among countries. Random-effect models were performed by R software (version 3.6.3), and sensitivity analysis was conducted via SPSS 25.

## Results

**Fig. 1:**
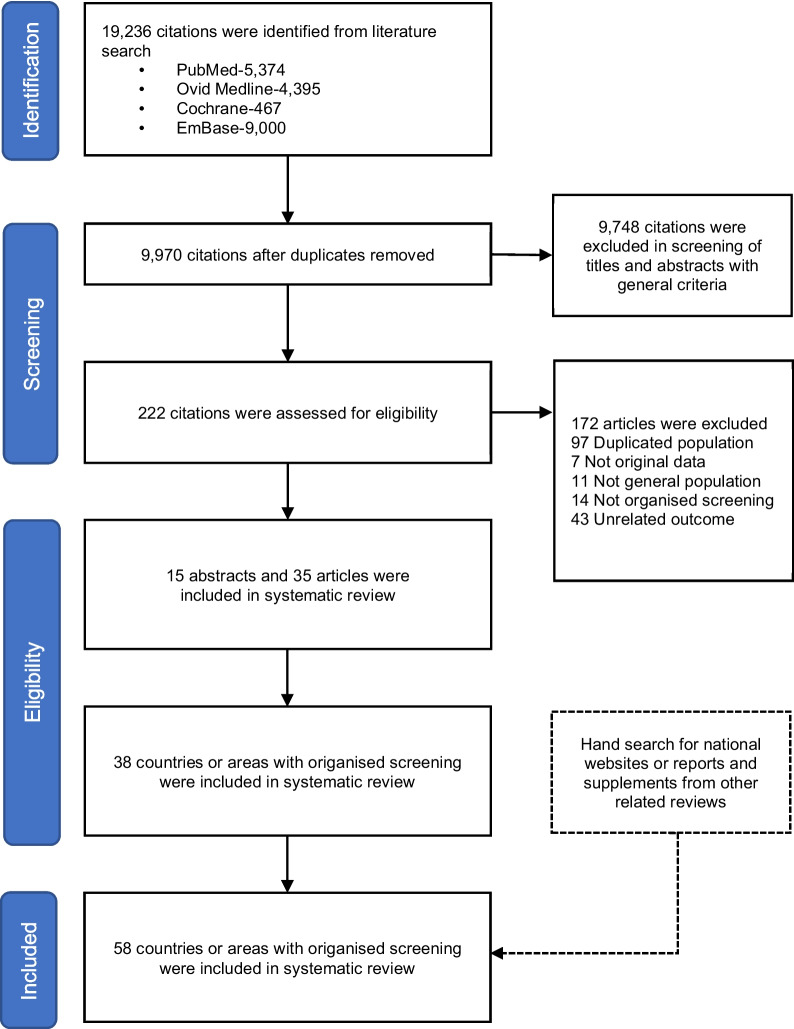
Flow diagram of the systematic review

A total of 19,236 citations were identified from the literature search. A sum of 9,970 citations remained after the removal of duplicates, and 9,748 citations were further excluded in the first stage of screening. After the second stage of screening, 38 countries or areas were included from 15 abstracts and 35 articles. After hand search for national websites or reports and supplements from other related reviews, a total of 58 countries or areas with organised screening were included in this study (Fig. 1). The characteristics of these organised CRC screenings were shown in Table S2.

Among countries with the long-term implementation of CRC screening, Belgium, Czech, Finland, France, Portugal, Spain, Sweden, UK (including England, Scotland and Wales), and Israel had changed gFOBT to FIT as their primary screening test. Germany and Poland adopted colonoscopy as the primary screening test for all eligible participants, while Czech and Israel used colonoscopy as an alternative option together with faecal tests for screening. Due to the different screening phases of the index year, the range of coverage rates and participation rates varied widely.

**Fig. 2:**
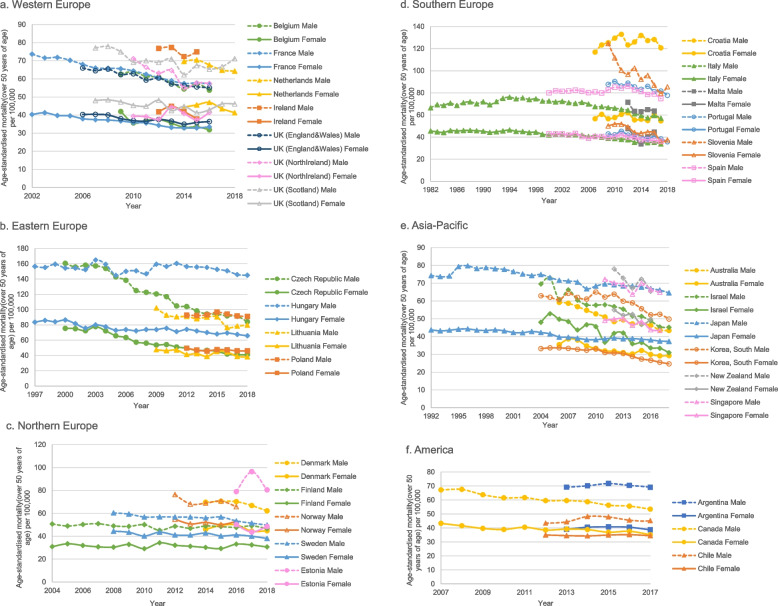
Changes in age-standardised mortality among countries that performed organised colorectal cancer screening

A total of 31 countries or areas can be matched with the cancer mortality database of the IARC, and the age-standardised mortality change (over 50 years of age) was presented in Fig. 2. Countries with the long-term implementation of screening programmes (e.g., France, Czech, Australia, Israel, and Canada) showed a decreasing mortality trend. However, there was no mortality reduction for countries (e.g., Estonia, Hungary, and Finland) where CRC screening was implemented for less than five years or was conducted in the pilot phase.

**Fig. 3:**
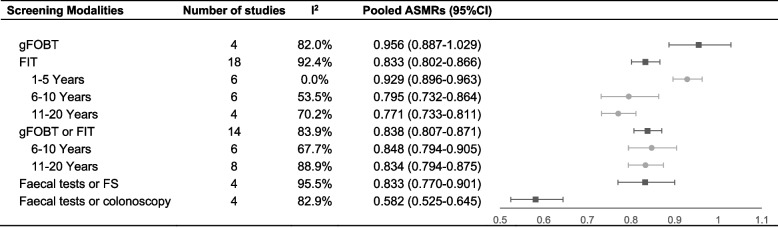
The pooled age-standardised colorectal cancer-related mortality ratio by screening modalities and duration. ASMR: age-standardised mortality ratio, gFOBT: guaiac faecal occult blood test, FIT: faecal immunochemical tests, FS: flexible sigmoidoscopy. Each country had two studies (male and female), and this table only showed subgroups with at least two countries or areas (four studies). gFOBT subgroup involved Croatia and the UK (Scotland) since the most available year of mortality in Scotland is 2017. FIT subgroup involved Australia, Ireland, Japan, South Korea, Lithuania, Malta, Singapore, Slovenia, and the Netherlands. gFOBT and FIT subgroup involved Belgium, Canada, France, Portugal, Spain, Sweden, and the UK (North Ireland). Faecal tests and FS subgroup involved Italy and the UK (England&Wales). Faecal tests and colonoscopy subgroup involved Czech Republic and Israel

After excluding countries or areas with pilot programmes, we finally obtained more than 2.7 million CRC-related deaths from 22 countries or areas. The programmes that offered a choice of screening modality between faecal tests and colonoscopy (pooled ASMR = 0.582, 95% CI = 0.525–0.645, I^2^ = 82.9%) was associated with better performance as compared to gFOBT (pooled ASMR = 0.956, 95% CI = 0.887–1.029, I^2^ = 82.0%), FIT (pooled ASMR = 0.833, 95% CI = 0.802–0.866, I^2^ = 92.4%), gFOBT or FIT (pooled ASMR = 0.838, 95% CI = 0.807–0.871, I^2^ = 83.9%) and faecal tests or FS (pooled ASMR = 0.833, 95%CI = 0.770–0.901, I^2^ = 95.5%) as the primary screening test. Among the FIT screening subgroup, the pooled ASMRs were 0.929 (95% CI = 0.896–0.963, I^2^ = 0.0%), 0.795 (95% CI = 0.732–0.864, I^2^ = 53.5%), and 0.771 (95% CI = 0.733–0.811, I^2^ = 70.2%) for 1–5 years, 6–10 years, and 11–20 years after screening implementation, respectively. The pooled ASMRs showed similar reducing trend with longer time of implementation for gFOBT and FIT subgroup (0.848 for 6–10 years and 0.834 for 11–20 years) (Fig. 3).

**Fig. 4:**
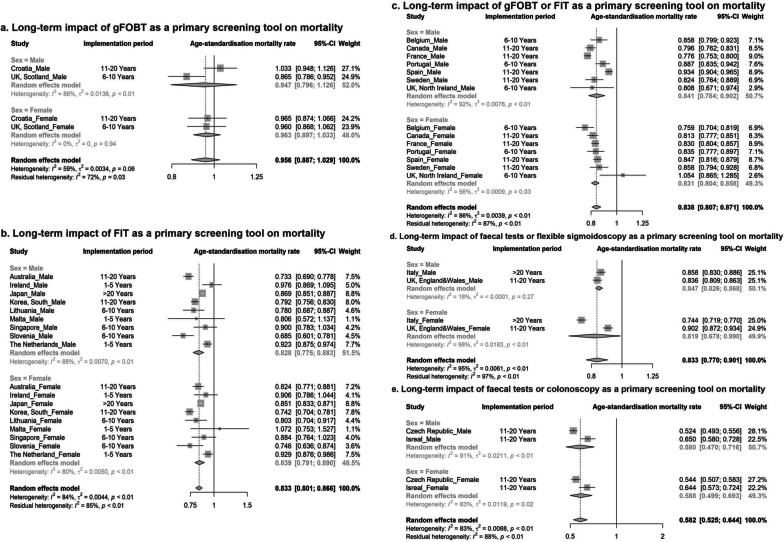
The pooled age-standardised colorectal cancer-related mortality ratio by screening modalities and sexes

Furthermore, our results showed that FIT only, gFOBT or FIT, faecal tests or FS, and faecal tests or colonoscopy as the primary screening test had significant associations with decreased mortality in both sexes (ASMRs: 0.580–0.847). However, the ASMRs for gFOBT only were non-significant in males (0.947, 95%CI = 0.796–1.126, I^2^ = 86%) and females (0.963, 95%CI = 0.897–1.033, I^2^ = 0%) (Fig. 4).

According to the linear mixed model, the screening strategy with faecal test or colonoscopy as a primary screening tool showed a significant decrease in ASMR (P = 0.004) when compared with gFOBT. The other screening strategies did not significantly differ from gFOBT as a primary test.

## Discussions

In this study, we analysed the characteristics of organised CRC screening programmes that have been performed in 58 countries or areas worldwide. The screening using faecal tests or colonoscopy as the primary screening test was associated with a greater reduction in mortality, followed by faecal tests or FS, FIT only, gFOBT or FIT, and gFOBT only. Moreover, the CRC-related morality was also influenced by screening duration. The pooled ASMRs of mortality reduction in the FIT screening subgroup showed a decreasing trend with a longer duration of screening implementation. Additionally, we found that programmes with gFOBT only as a primary screening test had a limited impact on mortality reduction in males and females. The sensitivity analysis also supported our result that screening strategy offers faecal test or colonoscopy as a primary screening test showed a significant reduction in ASMR in comparison with gFOBT.

Existing evidence suggests that FIT had better performance than gFOBT due to the higher participation rate and higher detection rate of CRC [12, 13]. Our study indicated that the organised screening with the FIT was associated with a greater reduction in mortality (16.7%) than those with gFOBT (4.4%) as the primary screening test, which was consistent with previous research. A network meta-analysis reported that FIT reduced 59% of CRC-related mortality, while gFOBT only reduced CRC mortality by 14% [14]. Gini and colleagues reported about 8–16% of the decline in mortality attributable to gFOBT and 35–41% of mortality decline due to FIT screening in Europe [15]. The differences in mortality reduction between our study and others might be explained by the variations in screening coverage, screening uptake, people’s attitude to CRC screening, development of novel treatment strategies, and lifestyle modification [16].

Besides these two screening strategies, colonoscopy was found to be associated with a 61–68% decline in CRC-related mortality [14, 17, 18]. However, few countries or areas are equipped with adequate resources to offer colonoscopy as a primary test in CRC screening programmes. Furthermore, some countries offered colonoscopy as an alternative option for a primary screening test. We reported that the screening with faecal tests or colonoscopy as a primary screening test had the best performance (42%) among CRC screening modalities. Among this subgroup, the Czech adopted faecal tests or colonoscopy for eligible participants aged above 50 years, while colonoscopy alone was offered to the high-risk population in Israel [19, 20]. A tailor-made screening strategy by risk was recommended by the Asia–Pacific Working Group on Colorectal Cancer, and they adopted the Asia–Pacific Colorectal Screening score to stratify individuals according to the risk of advanced neoplasia [21].

CRC screening programmes tend to have a time lag to observe the benefit, as it often takes years for symptoms to appear or before death occurs. The United States Preventive Services Task Force indicated that it should take at least seven years to observe the benefit of CRC screening [22]. Another survival meta-analysis estimated that it took about 4.8 years to prevent one CRC-related death per 5000 participants screened [23]. In our study, we found a non-significant ASMR of mortality reduction in screening programmes implemented with a duration of less than five years, which may imply the existence of the time lag effect of CRC screening. However, when the duration of screening implementation exceeded five years, we found that all subgroups had significant ASMRs of mortality decrease and that the length of duration was significantly associated with lower mortality. A longer duration of screening might extend a larger screening coverage and have a higher uptake rate, which could be translated to a more obvious mortality reduction in the general population.

Owing to the consideration of the programme surveillance and quality control, we only included organised screening in this study. Opportunistic screening requires a well-developed primary care system that may contribute to a reduction in CRC-related mortality, for example, in the US, where had fallen its CRC-related mortality in these two decades. However, it may reduce the cost and gain more effectiveness if organised screening is adopted. Although there is no cost-effectiveness analysis (CEA) in the US to compare the outcomes of organised and opportunistic CRC screening programmes, there was a CEA that supported organised cervical cancer screening in another developed area (Hong Kong) [24]. Moreover, organised screening could help to remain the equity of access in cancer screening [25]. Also, organised screening has a higher uptake rate than opportunistic screening. For example, Germany implemented the opportunistic screening programme and introduced colonoscopy as a primary screening test, but the participation rate was suboptimal [26]. Researchers suggested introducing organised screening to replace opportunistic screening to improve adherence [27, 28].

This study has some strengths. This study firstly reviewed the long-term impact of all organised CRC screening programmes on CRC-related mortality from a global perspective. In addition, we extracted the mortality of people over 50 years of age rather than the general population, as most CRC screening was initiated at the age of 50 years, which is more appropriate to observe the benefit of CRC screening. Moreover, we also observed the mortality changing in relation to the screening duration with the time lag effect of mortality. Also, the linear mixed model was used in this study to test the robustness as a sensitivity analysis. Nevertheless, some limitations should be addressed. First, our mortality reduction rates are different from that of previous studies, which may be due to the existence of potential confounders. These include people’s attitude to CRC screening, changes in lifestyle habits over time, and the development of novel cancer treatments that were not able to be accounted for, although a study from the US suggested that only 12% of mortality decline was attributed to treatment [29]. Second, the rollout process and uptake were not controlled for in this study, hence we excluded the pilot programmes from pooled estimates and conducted subgroup analyses according to the duration of screening implementation.

In conclusion, CRC screening programmes with a duration of implementation for over 5 years had an association with CRC-related mortality reduction, and the primary screening strategy which provides an option of faecal tests vs. colonoscopy is recommended for countries with adequate health resources. As the duration of programme implementation plays a role, we suggest formulation of a sustainable, organised CRC screening programme as soon as possible in countries where the burden of cancer is likely to increase substantially in the near future.

### Supplementary Information

**Supplementary Material 1.**

## Data Availability

All studies involved in this systematic review were in Supplementary Table 2. The matched mortality data were retrieved from the International Agency for Research on Cancer https://gco.iarc.fr/overtime/en.

## Abbreviations

CRC: Colorectal cancer
gFOBT: guaiac Faecal occult blood test
FIT: Faecal immunochemical test
FS: Flexible sigmoidoscopy
IARC: International Agency for Research on Cancer
ASMR: Age-standardised mortality ratio
CI: Confidence interval
CEA: Cost-effectiveness analysis
