# Supplementary material for: The association between organised colorectal cancer screening strategies and reduction of its related mortality: a systematic review and meta-analysis
Source: BMC Cancer. 2024 Mar 21;24:365. doi: 10.1186/s12885-024-12054-7 (PMC10958856; doi:10.1186/s12885-024-12054-7)
Supplement: Supplementary file 1 — Supplementary Material 1. [file 12885_2024_12054_MOESM1_ESM.docx]

**Supplementary Table 1. Search strategy and process**

| **PubMed** | **Query** | **Results** |
| --- | --- | --- |
| 1 | colorectal[Title/Abstract] OR bowel[Title/Abstract] OR colon[Title/Abstract] OR rectal[Title/Abstract] | 486,728 |
| 2 | cancer[Title/Abstract] OR tumor[Title/Abstract] OR tumour[Title/Abstract] OR neoplasm[Title/Abstract] OR adenoma[Title/Abstract] OR carcinoma[Title/Abstract] | 2,901,507 |
| 3 | screening[Title/Abstract] OR "Early Detection of Cancer"[Title/Abstract] | 545,010 |
| 4 | mass[Title/Abstract] OR general[Title/Abstract] OR organised[Title/Abstract] OR organized[Title/Abstract] OR national[Title/Abstract] | 2,401,154 |
| 5 | #1 AND #2 AND #3 AND #4 | ***5,374*** |
| **Ovid Medline** | **Searches** | **Results** |
| 1 | ('colorectal' or 'bowel' or 'colon' or 'rectal').ab,ti. | 415,813 |
| 2 | ('cancer' or 'neoplasm' or 'tumor' or 'tumour' or 'adenoma' or 'carcinoma').ab,ti. | 2,463,895 |
| 3 | ('mass' or 'general' or 'organised' or 'organized' or 'national').ab,ti. | 1,976,733 |
| 4 | ('screening' or 'early detection of cancer').ab,ti. | 456,745 |
| 5 | #1 AND #2 AND #3 AND #4 | ***4,395*** |
| **Embase** | **Searches** | **Results** |
| 1 | 'colorectal':ab,ti OR 'bowel':ab,ti OR 'colon':ab,ti OR 'rectal':ab,ti | 715,380 |
| 2 | 'cancer':ab,ti OR 'neoplasm':ab,ti OR 'carcinoma':ab,ti OR 'tumor':ab,ti OR 'tumour':ab,ti OR 'adenoma':ab,ti | 3,936,595 |
| 3 | 'mass':ab,ti OR 'general':ab,ti OR 'national':ab,ti OR 'organised':ab,ti OR 'organized':ab,ti | 3,139,897 |
| 4 | 'screening':ab,ti OR 'early detection of cancer':ab,ti | 755,530 |
| 5 | #1 AND #2 AND #3 AND #4 | ***9,000*** |
| **Cochrane** | **Searches** | **Results** |
| 1 | MeSH descriptor: [Mass Screening] explode all trees | 3,767 |
| 2 | MeSH descriptor: [Colorectal Neoplasms] explode all trees | 8,197 |
| 3 | #1 AND #2 | ***467*** |

**Supplementary Table 2. The characteristics of national or regional organised colorectal cancer screening**

| **Countries** | **Regions** | **Current**  **Status** | **Age range** | **Modalities** | **Interval time** | **Starting year** | **Male** | | **Female** | | **Index year** | **Reference** |
| --- | --- | --- | --- | --- | --- | --- | --- | --- | --- | --- | --- | --- |
|  |  |  |  |  |  |  | **Coverage** | **Participation** | **Coverage** | **Participation** |  |  |
| Belgium | Wallonia & Brussels | Rollout ongoing | 50–74 | gFOBT (2009-2016),  FIT (2016-) | 2 | 2009 | 101.6% | 6.3% | 96.5% | 6.9% | 2014 | [1] |
| Belgium | Flanders | Rollout ongoing | 56–74 | FIT | 2 | 2013 | 100.5% | 46.9% | 98.3% | 49.5% | 2014 | [1] |
| Croatia | All | Rollout complete | 50–74 | gFOBT | 2 | 2007 | 100.5% | 15.2% | 100.5% | 15.2% | 2013-2014 | [1–3] |
| Cyprus | All | Pilot | 50-69 | FIT | 2 | 2013 |  |  |  |  |  | [4] |
| Czech Republic | All | Rollout ongoing | 50- | gFOBT (2000-2009), FIT (2009-), CS (2009-) | 1/2 (gFOBT, FIT), 10 (CS) | 2000 | 53.0% | 17.3% | 53.0% | 17.3% | 2014 | [1, 5, 6] |
| Denmark | All | Rollout ongoing | 50–74 | FIT | 2 | 2014 |  | 58.4% |  | 58.4% | 2014-2016 | [7] |
| Estonia | All | Pilot | 60-69 | FIT | 2 | 2016 |  | 43.7% |  | 43.7% | 2016-2018 | [8] |
| Finland | All | Pilot | 60–69 | gFOBT (2004-2016),  FIT (2017-) | 2 | 2004 | 24.2% | 59.1% | 23.7% | 73.4% | 2014 | [1, 9, 10] |
| France | All | Rollout complete | 50–74 | gFOBT (2002-2015),  FIT (2015-) | 2 | 2002 | 100.0% | 29.0% | 100.0% | 33.0% | 2012-2013 | [11–13] |
| Germany | Regions | Rollout ongoing | 50 (Male),  55 (Female) | CS | 9 | 2019 |  |  |  |  |  | [14] |
| Hungary | Regions | Pilot | 50-69 | FIT | 2 | 1997 | 1.8% | 33.3% | 1.7% | 41.2% | 2013 | [1, 15, 16] |
| Ireland | All | Rollout ongoing | 60-69 | FIT | 2 | 2012 | 28.6% | 40.2% | 28.6% | 40.2% | 2013 | [1, 17, 18] |
| Italy | All | Rollout ongoing | 50-74 | FIT (1982-), FS (2003-) | 2, once (FS) | 1982 | 63.0% | 45.4% | 63.0% | 45.4% | 2013 | [1, 19] |
| Lithuania | All | Rollout ongoing | 50-74 | FIT | 2 | 2009 |  | 47.0% |  | 57.5% | 2014 | [1, 20] |
| Luxembourg | All | Rollout ongoing | 55-74 | FIT | 2 | 2016 |  |  |  |  |  | [1, 21] |
| Malta | All | Rollout ongoing | 55-66 | FIT | 2 | 2012 | 127.1% | 35.7% | 127.1% | 35.7% | 2014 | [1, 22] |
| Montenegro | All | Rollout ongoing | 59-64 | Questionnaire and FOBT |  | 2013 |  |  |  |  |  | [21] |
| The Netherlands | All | Rollout ongoing | 55-75 | FIT | 2 | 2014 | 95.2% | 70.6% | 95.2% | 74.8% | 2017 | [23–25] |
| Norway | Regions | Pilot | 50-74 | FIT, FS | 2 | 2012 |  |  |  |  |  | [26] |
| Poland | All | Pilot^a^ | 55-64 | CS | 10 | 2012 | 9.8% | 18.4% | 10.4% | 15.4% | 2013 | [1] |
| Portugal | Regions | Rollout ongoing | 50-74 | gFOBT (2009-2018),  FIT (2012-) | 2 | 2009 | 1.8% | 61.1% | 1.8% | 61.1% | 2014 | [1] |
| San Marino | All | Rollout complete | 50-75 | FIT | 2 | 2009 | 80.0% | 60.0% | 80.0% | 60.0% | 2009-2014 | [27] |
| Serbia | Regions | Pilot | 50-74 | FIT | 2 | 2013 | 19.0% | 62.5% | 19.0% | 62.5% | 2013-2014 | [28] |
| Slovenia | All | Rollout complete | 50-74 | FIT | 2 | 2009 | 93.0% | 45.7% | 93.8% | 55.1% | 2011-2012 | [1, 29, 30] |
| Spain | All | Rollout ongoing | 50-69 | gFOBT (2000-2010),  FIT (2010-) | 2 | 2000 | 16.0% | 48.8% | 16.8% | 51.8% | (FIT) 2013 | [1, 31] |
| Sweden | Stockholm-Gotland | Rollout ongoing | 60-69 | gFOBT (2008-2015),  FIT (2015-) | 2 | 2008 | 19.3% | 54.9% | 20.4% | 64.7% | (gFOBT) 2013 | [1, 32] |
| UK | England | Rollout complete | 60-74,  55-59 (FS) | gFOBT (2006-2018),  FS (2013-2021),  FIT (2018-) | 2 | 2006 | 94.8% | 52.8% | 104.2% | 57.7% | 2013 | [1, 19, 33] |
| UK | Scotland | Rollout complete | 50-74 | gFOBT (2007-2017),  FIT (2017-) | 2 | 2007 | 112.1% | 54.3% | 108.6% | 59.5% | 2013 | [1, 19, 34] |
| UK | Wales | Rollout complete | 60-74 | gFOBT (2008-2019),  FIT (2019-) | 2 | 2008 | 89.3% | 58.3% | 88.3% | 63.9% | 2013 | [1, 19] |
| UK | Northern Ireland | Rollout complete | 60-74 | gFOBT or FIT | 2 | 2010 | 98.1% | 54.6% | 98.1% | 54.6% | 2013 | [1, 19] |
| Argentina | Urban areas | Pilot |  |  |  | 2013 |  |  |  |  |  | [35] |
| Brazil | Regions | Pilot | 50-75 | FIT |  |  |  |  |  |  |  | [19] |
| Canada | Ontario |  | 50-74 | gFOBT | 2 | 2008 | 100.0% |  | 100.0% |  | 2013-2014 | [36] |
| Canada | British Columbia |  | 50-74 | FIT | 2 | 2009 | 100.0% |  | 100.0% |  | 2013-2014 | [36] |
| Canada | Alberta |  | 50-74 | gFOBT (2007-2013), FIT (2013-) | 1 or 2 | 2007 | 100.0% | 41.8% | 100.0% | 41.8% | 2013-2014 | [36] |
| Canada | Saskatchewan |  | 50-74 | FIT | 2 | 2009 | 100.0% | 53.0% | 100.0% | 53.0% | 2013-2014 | [36] |
| Canada | Manitoba |  | 50-74 | gFOBT | 2 | 2007 | 100.0% | 16.9% | 100.0% | 16.9% | 2013-2014 | [36] |
| Canada | New Brunswick |  | 50-74 | FIT | 2 | 2014 |  | 25.8% |  | 25.8% | 2013-2014 | [36] |
| Canada | Nova Scotia |  | 50-74 | FIT | 2 | 2009 | 100.0% |  | 100.0% |  | 2013-2014 | [36] |
| Canada | Prince Edward Island |  | 50-74 | FIT | 2 | 2009 | 100.0% | 20.4% | 100.0% | 20.4% | 2013-2014 | [36] |
| Canada | Newfoundland and Labrador |  | 50-74 | FIT | 2 | 2012 | 10-49% | 8.6% | 10-49% | 8.6% | 2013-2014 | [36] |
| Costa Rica | Cartago | Pilot | 50-74 | FIT |  | 2017 |  | 40.0% |  | 40.0% | 2017-2019 | [37] |
| Chile | Regions | Pilot | 50-75 | FIT |  | 2012 |  |  |  |  |  | [19, 38] |
| Martinique | All | Rollout complete | 50–74 | FIT |  | 2007 |  |  |  |  |  | [19] |
| USA | Northern and Southern California |  | 51-75 | FIT | 1 | 2007 |  | 82.7%^c^ |  | 82.7%^c^ | 2015 | [39] |
| Australia | All | Rollout complete | 50-74 | FIT | 2 | 2006 |  | 39.4% |  | 43.2% | 2016-2017 | [40] |
| China | 16 provinces | Pilot | 40-69 | Questionnaire |  | 2012 |  |  |  |  |  | [41] |
| China | Hong Kong | Rollout ongoing | 56-75 | FIT | 2 | 2016 | 16.6% | 96.8% | 16.6% | 96.8% | 2016-2019 | [42] |
| China | Taiwan | Rollout ongoing | 50-69 | FIT | 2 | 2004 | 36.9% | 65.5% | 36.9% | 65.5% | 2010-2013 | [43] |
| Israel | All | Rollout complete | 50-74 | gFOBT (2004-2012), FIT (2012-), CS (High risk) | 1 (gFOBT, FIT), 10 (CS) | 2004 |  | 64.7% |  | 64.7% | 2018 | [44, 45] |
| Japan | All | Rollout complete | 40-69 | FIT |  | 1992 |  | 41.4% |  | 34.5% | 2013 | [46, 47] |
| Kazakhstan |  | Pilot | 50-70 | FIT | 2 | 2011 |  |  |  |  |  | [48] |
| Korea, South | All | Rollout complete | 50- | FIT | 1 | 2004 |  | 43.4-45.0% |  | 55.0-56.6% | 2004-2007 | [49, 50] |
| New Zealand | All | Rollout ongoing^b^ | 50-74 (2012-2018), 60-74 (2017-) | FIT |  | 2012 |  | 61.1% |  | 64.7% | 2015 | [51] |
| Qatar |  | Pilot | 50-74 | FIT | 2 | 2016 |  |  |  |  |  | [52] |
| Singapore | All | Rollout complete | 50- | FIT | 1 | 2011 |  |  |  |  |  | [53] |
| Thailand |  | Pilot | 50–70 | FIT |  | 2017 |  | 57.8% |  | 67.8% | 2011-2012 | [54] |
| Algeria | Béjaia | Pilot | 50-74 | FIT |  | 2015 |  | 26.0% |  | 26.0% | 2016-2017 | [55] |

gFOBT: guaiac faecal occult blood test, FIT: faecal immunochemical test, FS: flexible sigmoidoscopy, CS: colonoscopy. #: The pilot programme was conducted via randomised health services in Poland. * The pilot programme was performed from 2012-2018 in Waitematā and the target population aged 50-74 years. ^ Extracted from cohort of Kaiser Permanente Northern California.

**Table reference**

1. Ponti A, Anttila A, Ronco G, Senore C. Cancer Screening in the European Union (2017) Report on the implementation of the Council Recommendation on cancer screening. 2017.

2. Antoljak N, Šekerija M. Epidemiology and screening of colorectal cancer. Libr Oncol. 2013;41:3–8.

3. Kalauz M, Antoljak N, Katiĉić M, Skoko Poljak D, Stamenić V, Stimac D, et al. Second reorganised cycle of national colorectal cancer screening programme in croatia. United Eur Gastroenterol J. 2016;4:A278–9.

4. United European Gastroenterology. COLORECTAL SCREENING ACROSS EUROPE. Vienna.

5. Suchanek S, Grega T, Majek O, Ngo O, Seifert B, Dusek L, et al. Population-based colorectal cancer screening in the Czech Republic-first results of the new program settings. United Eur Gastroenterol J. 2017;5:A770–1.

6. Suchanek S, Majek O, Vojtechova G, Minarikova P, Rotnaglova B, Seifert B, et al. Colorectal cancer prevention in the Czech Republic: Time trends in performance indicators and current situation after 10 years of screening. Eur J Cancer Prev. 2014;23:18–26.

7. Njor SH, Friis-Hansen L, Andersen B, Søndergaard B, Linnemann D, Jørgensen JCR, et al. Three years of colorectal cancer screening in Denmark. Cancer Epidemiol. 2018;57:39–44.

8. Eisen M, Tomberg K, Innos K. Colorectal cancer screening: The first three years experience of national colorectal cancer screening programme in Estonia. Dig Endosc. 2020;32:143.

9. Finnish Cancer registry. New strategy for colorectal cancer screening in Finland Background. Helsinki; 2017.

10. Malila N, Palva T, Malminiemi O, Paimela H, Anttila A, Hakulinen T, et al. Coverage and performance of colorectal cancer screening with the faecal occult blood test in Finland. J Med Screen. 2011;18:18–23.

11. Assogba F, Jezewski-Serra D, Lastier D, Quintin C. Outcomes of the French colorectal cancer population-based screening programme using guaiac faecal occult blood test. United Eur Gastroenterol J. 2014;2:A99–100.

12. Barré S, Leleu H, Vimont A, Kaufmanis A, Gendre I, Taleb S, et al. [Estimated impact of the current colorectal screening program in France]. Rev Epidemiol Sante Publique. 2020;68:171–7.

13. Goulard H, Boussac-Zarebska M, Ancelle-Park R, Bloch J. French colorectal cancer screening pilot programme: results of the first round. J Med Screen. 2008;15:143–8.

14. Gemeinsamer Bundesausschuss. Richtlinie Für Organisierte Krebsfrüherkennungsprogramme [Directive On Organised Cancer Screening Programmes]. https://www.g-ba.de/richtlinien/104/. Accessed 25 Jun 2021.

15. Kívés Z, Juhász K, Csákvári T, Ágoston I, Endrei D. Cancer screening policy in Hungary. Int J Cancer. 2018;143:1003–4.

16. Simon J, Galasz V, Antal I. Evaluation of the laboratory method of public health screening program for colorectal cancer screening in Hungary. Clin Chem Lab Med. 2016;54:eA199.

17. Fahy L, Fitzpatrick P, Meade C, Farrell HC, O’Donoghue D, Mooney T. Impact of the introduction of a new policy of direct faecal immunochemical home screening test provision in a national bowel screening programme, both during and outside of advertising campaigns. Cancer Epidemiol. 2020;69:101844.

18. O’Donoghue D, Sheahan K, MacMathuna P, Stephens RB, Fenlon H, Morrin M, et al. A National Bowel Cancer Screening Programme using FIT: Achievements and Challenges. Cancer Prev Res. 2019;12:89–94.

19. Schreuders EH, Ruco A, Rabeneck L, Schoen RE, Sung JJY, Young GP, et al. Colorectal cancer screening: a global overview of existing programmes. https://doi.org/10.1136/gutjnl-2014-309086.

20. Poskus T, Strupas K, Mikalauskas S, Bitinaite D, Kavaliauskas A, Samalavicius NE, et al. Initial results of the National Colorectal Cancer Screening Program in Lithuania. Eur J Cancer Prev. 2015;24:76–80.

21. Young GP, Rabeneck L, Winawer SJ. The Global Paradigm Shift in Screening for Colorectal Cancer. Gastroenterology. 2019;156:843-851.e2.

22. Gabriella Buttigieg, Rachel Abela. A Retrospective study on the National Colorectal Screening Programme: Analysis of participation and findings. THESYNAPSE.net. 2018;17:6–8.

23. Nederlands kanker Instituut. Monitoring and Evaluation of the Colorectal Cancer Screening Programme 2017. 2018.

24. Spaander M, Wisse P, Boer SYD, Hartog BD, Pool MO, Terhaar sive Droste JS, et al. COLONOSCOPY QUALITY ASSURANCE IN AN ORGANIZED FIT-BASED COLORECTAL CANCER SCREENING PROGRAM. Gastrointest Endosc. 2020;91:AB463–4.

25. Toes-Zoutendijk E, van Leerdam ME, Dekker E, van Hees F, Penning C, Nagtegaal I, et al. Real-Time Monitoring of Results During First Year of Dutch Colorectal Cancer Screening Program and Optimization by Altering Fecal Immunochemical Test Cut-Off Levels. Gastroenterology. 2017;152:767-775.e2.

26. Kaminski MF, Kraszewska E, Rupinski M, Laskowska M, Wieszczy P, Regula J. Design of the Polish Colonoscopy Screening Program: A randomized health services study. Endoscopy. 2015;47:1144–50.

27. Piscaglia AC, Calò G, Gerardi V, Laterza L, Sacchini E, Fantini GF, et al. Colorectal cancer screening program in San Marino: A small republic with a screening model of high quality. Dig Liver Dis. 2015;47:e92.

28. Banković Lazarević D, Krivokapić Z, Barišić G, Jovanović V, Ilić D, Veljković M. Organized colorectal cancer screening in Serbia - the first round within 2013-2014. Vojn Pregl. 2016;73:360–7.

29. Novak Mlakar D, Kofol Bric T. Slovenian national colorectal cancer screening – Programme SVIT. Eur J Public Health. 2018;28 suppl_4.

30. Tepeš B, Bracko M, Novak Mlakar D, Stefanovic M, Stabuc B, Frkovic Grazio S, et al. Results of the FIT-based National Colorectal Cancer Screening Program in Slovenia. J Clin Gastroenterol. 2017;51:e52–9.

31. Benito L, Travier N, Binefa G, Vidal C, Espinosa J, Milà N, et al. Longitudinal Adherence to Immunochemical Fecal Occult Blood Testing vs Guaiac-based FOBT in an Organized Colorectal Cancer Screening Program. Cancer Prev Res. 2019;12:327–34.

32. Saraste D, Öhman DJ, Sventelius M, Elfström KM, Blom J, Törnberg S. Initial participation as a predictor for continuous participation in population-based colorectal cancer screening. J Med Screen. 2018;25:126–33.

33. Logan RF, Patnick J, Nickerson C, Coleman L, Rutter MD, von Wagner C. Outcomes of the Bowel Cancer Screening Programme (BCSP) in England after the first 1 million tests. Gut. 2012;61:1439–46.

34. Clark G, Strachan JA, Carey FA, Godfrey T, Irvine A, McPherson A, et al. Transition to quantitative faecal immunochemical testing from guaiac faecal occult blood testing in a fully rolled-out population-based national bowel screening programme. Gut. 2020. https://doi.org/10.1136/gutjnl-2019-320297.

35. Sierra MS, Forman D. Burden of colorectal cancer in Central and South America. Cancer Epidemiol. 2016;44:S74–81.

36. Canadian Partnership Against Cancer. Colorectal Cancer Screening in Canada: Monitoring & Evaluation of Quality Indicators – Results Report, January 2013 – December 2014. Toronto; 2017.

37. Cabo AS. First organized colorectal cancer screening in Costa Rica: Results from the initial round of screening for the province of cartago. Dig Endosc. 2020;32:21–2.

38. Kobayashi M, Kawachi H, Pasternak S, Delgado C, Pinto P, Ito T, et al. Histopathologic study from a colorectal cancer screening in Chile: results from the first 2 years of an international collaboration between Chile and Japan. Eur J Cancer Prev. 2019;28:245–53.

39. Levin TR, Corley DA, Jensen CD, Schottinger JE, Quinn VP, Zauber AG, et al. Effects of Organized Colorectal Cancer Screening on Cancer Incidence and Mortality in a Large Community-Based Population. Gastroenterology. 2018;155:1383-1391.e5.

40. Australian Institute of Health and Welfare. National Bowel Cancer Screening Program Monitoring report 2020. Canberra; 2020.

41. Chen H, Li N, Ren J, Feng X, Lyu Z, Wei L, et al. Participation and yield of a population-based colorectal cancer screening programme in China. Gut. 2019;68:1450–7.

42. Non-Communicable Disease Branch C for HPD of HHKSARG. Colorectal Cancer Screening Programme-Progress report of the screening outcome for participant enrolled between 28 September 2016 and 27 September 2019. Hong Kong; 2019.

43. Chou CK, Chen SLS, Yen AMF, Chiu SYH, Fann JCY, Chiu HM, et al. Outreach and inreach organized service screening programs for colorectal cancer. PLoS One. 2016;11.

44. The Israel Cancer Association. National Program for Early Detection of Colorectal Cancer . 2020. https://en.cancer.org.il/template_e/default.aspx?PageId=7747. Accessed 22 Jun 2021.

45. Paltiel O, Keidar Tirosh A, Paz Stostky O, Calderon-Margalit R, Cohen AD, Elran E, et al. Adherence to national guidelines for colorectal cancer screening in Israel: Comprehensive multi-year assessment based on electronic medical records. J Med Screen. 2020;28.

46. Sano Y, Byeon J-S, Li X-B, Wong MCS, Chiu H-M, Rerknimitr R, et al. Colorectal cancer screening of the general population in East Asia. Dig Endosc. 2016;28:243–9.

47. Saito H. Current status of colorectal cancer screening in Japan. Acta Endoscopica. 2007;37:181–8.

48. Balmagambetova S, Urazayev O, Tulyayeva A, Nurgaziyeva GM, Aitmagambetova M, Kereeva N, et al. Initial evaluation of the nationwide screening programs running in the Aktobe province of western Kazakhstan. Minerva Med. 2019;110:14–5.

49. Shin A, Choi KS, Jun JK, Noh DK, Suh M, Jung K-W, et al. Validity of Fecal Occult Blood Test in the National Cancer Screening Program, Korea. PLoS One. 2013;8:e79292.

50. Suh M, Song S, Cho HN, Park B, Jun JK, Choi E, et al. Trends in Participation Rates for the National Cancer Screening Program in Korea, 2002-2012. Cancer Res Treat. 2017;49:798–806.

51. Ministry of Health (New Zealand). Final Evaluation Report of the Bowel Screening Pilot Screening Rounds One and Two. Wellington; 2016.

52. John A, Varughese B, Pillai V, Singh R, Al Kaabi S. Colonoscopy outcomes in colorectal cancer screening population in Qatar. J Gastroenterol Hepatol. 2019;34:610.

53. Yong SK, Ong WS, Koh GC-H, Yeo RMC, Ha TC. Colorectal cancer screening: Barriers to the faecal occult blood test (FOBT) and colonoscopy in Singapore. Proc Singapore Healthc. 2016;25:207–14.

54. Khuhaprema T, Sangrajrang S, Lalitwongsa S, Chokvanitphong V, Raunroadroong T, Ratanachu-Ek T, et al. Organised colorectal cancer screening in Lampang Province, Thailand: preliminary results from a pilot implementation programme. BMJ Open. 2014;4.

55. Kamel B, Chahira M. Organized screening for colorectal cancer in Algeria: First pilot study in North Africa. J Clin Oncol. 2018;36.
